# Accuracy and postoperative assessment of robot-assisted placement of pedicle screws during scoliosis surgery compared with conventional freehand technique: a systematic review and meta-analysis

**DOI:** 10.1186/s13018-024-04848-z

**Published:** 2024-06-20

**Authors:** Wei Cui, Xinglin Liu, Zhiheng Zhao, Zihe Feng, Xianglong Meng

**Affiliations:** 1 Department of Orthopedic Surgery, Beijing AnZhen Hospital, Capital Medical University, No. 2 Anzhen Road, Chaoyang District, Beijing, 100029 China; 2 Department of Orthopedic Surgery, Beijing Chaoyang Hospital, Capital Medical University of China, S Sanlitun Rd, Chaoyang District, Beijing, 100020 China

**Keywords:** Scoliosis, Robotic, Robot-assisted surgical procedures, Freehand, Conventional, Pedicle screw

## Abstract

**Study design:**

A systematic review and meta-analysis.

**Background:**

The complexity of human anatomical structures and the variability of vertebral body structures in patients with scoliosis pose challenges in pedicle screw placement during spinal deformity correction surgery. Through technological advancements, robots have been introduced in spinal surgery to assist with pedicle screw placement.

**Methods:**

A systematic search was conducted using PubMed, Cochrane, Embase, and CNKI databases and comparative studies assessing the accuracy and postoperative efficacy of pedicle screw placement using robotic assistance or freehand techniques in patients with scoliosis were included. The analysis evaluated the accuracy of screw placement, operative duration, intraoperative blood loss, length of postoperative hospital stay, and complications.

**Results:**

Seven studies comprising 584 patients were included in the meta-analysis, with 282 patients (48.3%) in the robot-assisted group and 320 (51.7%) in the freehand group. Robot-assisted placement showed significantly better clinically acceptable screw placement results compared with freehand placement (odds ratio [OR]: 2.61, 95% confidence interval [CI]: 1.75–3.91, *P* < 0.0001). However, there were no statistically significant differences in achieving “perfect” screw placement between the two groups (OR: 1.52, 95% CI: 0.95–2.46, *P* = 0.08). The robot-assisted group had longer operation durations (mean deviation [MD]: 43.64, 95% CI: 22.25–64.74, *P* < 0.0001) but shorter postoperative hospital stays (MD: − 1.12, 95% CI: − 2.15 to − 0.08, *P* = 0.03) than the freehand group. There were no significant differences in overall complication rates or intraoperative blood loss between the two groups. There was no significant difference in Cobb Angle between the two groups before and after operation.

**Conclusion:**

Robot-assisted pedicle screw placement offers higher accuracy and shorter hospital stay than freehand placement in scoliosis surgery; although the robotics approach is associated with longer operative durations, similar complication rates and intraoperative blood loss.

**Supplementary Information:**

The online version contains supplementary material available at 10.1186/s13018-024-04848-z.

## Introduction

Scoliosis is a common three-dimensional (3D) deformity of the spine that affects multiple segments. Severe scoliosis not only affects the appearance but also leads to respiratory problems, trunk imbalance, and depression. When the angle of curvature exceeds 40°, brace treatment is ineffective, progression exceeds 5° per year, there is a significant visible deformity and surgical intervention becomes necessary [1]. Pedicle screw fixation is the most commonly used surgical method for treating scoliosis and provides patients with good deformity correction [2]. However, the inherent complexity of spinal structures, coupled with vertebral rotation and abnormal development of the pedicle roots in patients with scoliosis, such as asymmetrical pedicle sizes and lengths outside the normal physiological range, increases the difficulty of pedicle screw placement and leads to complications such as nerve damage [3–5].

Currently, freehand screw placement still dominates scoliosis correction surgery [6]. However, in related studies, the rate of freehand screws misplacement ranges from 1.5 to 43% [7]. Intraoperative navigation techniques, robot-assisted techniques, and augmented reality have been successively applied to reduce complications during spinal surgery and have improved the accuracy of pedicle screw placement. D’Souza et al. pointed out that robot-assisted screw placement is more accurate and has higher fusion rates than fluoroscopy-assisted surgery [8]. Furthermore, in a retrospective study conducted by Yu et al., the hospital stay of patients undergoing lumbar fusion surgery in the robot-assisted group was shorter than that in the freehand group (2.5 vs. 3.17 days, *P* < 0.018) [9]. In a multicenter study, Lee et al. found that patients undergoing lumbar fusion surgery with robot-assisted navigation technology had lower revision rates 1 year postoperatively [10].

Although numerous studies have shown that robot-assisted screw-placement technology in spinal surgery can improve accuracy, reduce complications, and shorten hospital stay, few studies have examined its application in scoliosis correction surgery, with contradictory results [11, 12]. Whether this technology can provide better postoperative outcomes than freehand screw placement in patients with scoliosis remains controversial. Therefore, this meta-analysis adopted a dual evaluation method of radiological and clinical assessment, summarized relevant data, and attempted to evaluate whether robot-assisted technology has higher screw placement accuracy and fewer complications in scoliosis correction surgery than freehand screw placement.

## Method

### Data search strategy

This meta-analysis adhered to the Preferred Reporting Items for Systematic Reviews and Meta-Analysis (PRISMA) 2020 statement [13]and was registered in PROSPERO with the identiffer CRD42024506367 before data extraction. Two reviewers (Wei Cui and Xinglin Liu) conducted a detailed systematic review on electronic databases using PubMed, Embase, Cochrane, and CNKI for articles published between January 1st, 1980 and November 1st, 2023. MeSH terms (using the Boolean operators “and” and “or”) which included “robotic surgery”, “scoliosis”, and “Pedicle Screws” were searched. All articles irrespective of the language were included in our study.

### Inclusion and exclusion criteria

Based on the PICOS principle, the criteria for inclusion and exclusion are as follows: (i) Participants: Patients with scoliosis undergoing pedicle screw placement surgery are included in this study.(ii) Intervention: Patients in the case group or experimental group undergo pedicle screw placement surgery assisted by robotic technology.(iii) Control: Patients in the control group undergo traditional free-hand screw placement methods.(iv) Outcome Measures: The primary endpoint is the accuracy of pedicle screw implantation, assessed using the Gertzbein-Robbins grading [14] or Mobbs-Raley grading [15]. Secondary endpoints include complications, operation time, length of hospital stay, and intraoperative blood loss. Our analysis only includes randomized controlled trials (RCTs), prospective cohort studies (PCS), and retrospective comparative studies (RCS). Case reports, technical reports, and case series are excluded.

### Data extraction and outcome measures

The data were extracted by the authors using a structured template form based on the Cochrane Consumers and Communication Group. We further conducted this meta-analysis in accordance with the criteria set by Cochrane Consumers and Communication Group reviews: Meta-analysis. Other authors will be invited to participate in the discussion until a consensus opinion is reached if there is disagreement about the outcome. From each study, the following data will be extracted: (i) Demographic characteristics, (ii) Clinical conditions, (iii) Robot type, (iv) Outcome measures (Accuracy of pedicle screw placement, Complications, Operation time, Intraoperative blood loss, Length of hospital stay).

### Evidence quality assessment

The meta-analysis included a total of 7 retrospective cohort studies [7, 11, 12, 16–19]. Two reviewers independently assessed the quality of evidence for each study using the Newcastle-Ottawa Scale (NOS) [20]. A higher score indicates better quality. The scale ranges from 0 to 9 stars, with studies scoring 7 stars or above considered to be of excellent quality.

### Statistical analysis

Review Manager (Rev Man 5.4) was used for comparing data from the included studies. Pooled weighted mean difference was used to analyze the continuous data, while the Odds Ratio (OR) was used to analyze the dichotomous data. The results were reported as either mean difference (MD) or odds ratio (OR) with a 95% confidence interval (CI). The heterogeneity among the studies was evaluated using I2 statistics. The fixed effect model was used for I2 < 50%, while for I2 > 50%, a random-effect model was employed. All tests were 2-tailed, and a *p* value of < 0.05 was considered statistically significant.

## Results

### Study selection

**Fig. 1:**
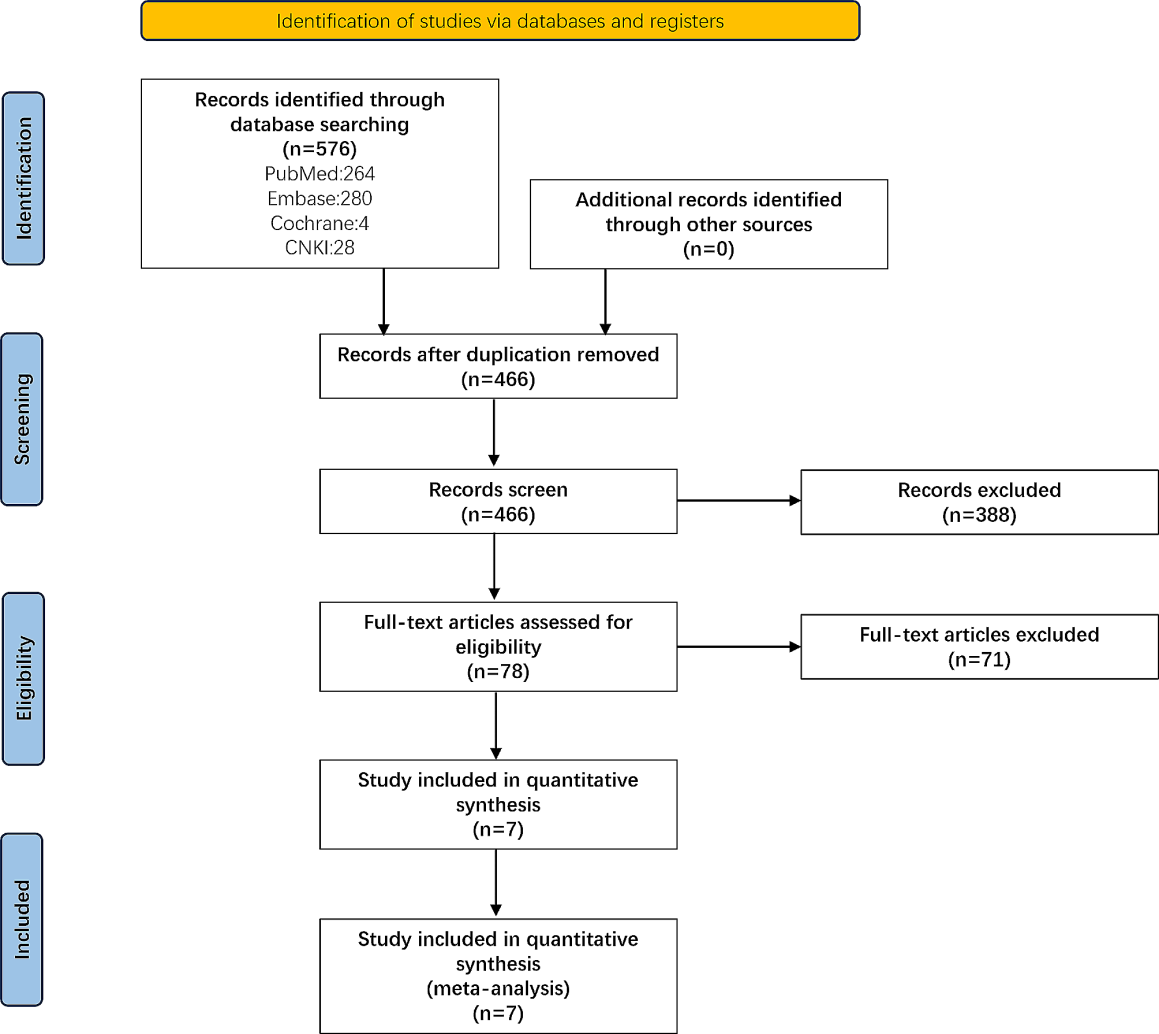
PRISMA flow chart for the search and inclusion strategy

From an initial search of electronic databases (PubMed, EMBASE, Cochrane, and CNKI), a total of 576 articles were retrieved. After excluding duplicate articles, 466 articles remained. These articles were reviewed according to the PRISMA guidelines (Fig. 1). Following abstract screening, 388 articles were excluded as they were not relevant to spinal deformity correction surgery. Based on the inclusion and exclusion criteria, a total of 78 full-text articles were assessed for eligibility, of which 71 articles were excluded. Therefore, 7 articles were included in our review, all of which were retrospective cohort studies (RCS). No prospective cohort studies (PCS) or randomized controlled trials (RCTs) were included.

### Overall characteristics of the study

Our analysis included a total of 584 patients, with 282 patients (48.3%, 2984 screws) in the intervention group and 320 patients (51.7%, 3729 screws) in the control group. The baseline characteristics of the included studies are shown in Table 1. The accuracy of pedicle screw placement was evaluated using postoperative thin-slice CT scans. The grading criteria used in the included studies included both the Gertzbein-Robbins and Mobbs-Raley grading systems. Although different grading criteria were used in the included studies, the criteria for perfect screw placement and clinically acceptable screw placement were consistent. Therefore, the use of different grading criteria did not affect the final result analysis. According to the NOS assessment, the evidence quality of 4 studies [11, 12, 16, 17] was considered excellent, while 3 studies [7, 18, 19] were considered fair (Table 2).

**Table 1:** Baseline characteristics of all the included studies

Author’s Name	Type of study	Level of Evidence	Country	Total No. of Patient	Pedicle Screws	Robot Type	Sex (M/F)	Age(y)	BMI
Canglong Hou et al. 2023	RCS	IIIb	China	RA 45 FH 56	RA 647 FH 771	Mazor	RA (13/32) FH (18/38)	RA(14.69 ± 1.93) FH (14.49 ± 2.01)	N/A
Chao Li et al. 2022	RCS	IIIb	China	RA 92 FH 52	RA 1080 FH 722	TianJi	RA (24/68) FH (17/35)	RA (32.2 ± 22.8) FH (29.1 ± 22.1)	RA (21.9 ± 2.8) FH (22.4 ± 3.1)
CHEN Haojie et al. 2021	RCS	IIIb	China	RA 22 FH 24	RA 343 FH 374	TianJi	RA (4/18) FH (5/19)	RA (13.9) FH (14.1)	RA (18.92 ± 1.01) FH (19.79 ± 1.95)
Gabriel S et al.2022	RCS	IIIb	USA	RA 30 FH 30	N/A	Mazor	RA (7/23) FH (7/23)	RA(15 ± 2.01) FH (15.3 ± 1.9)	N/A
LI Chao et al. 2023	RCS	IIIb	China	RA 44 FH 52	RA 230 FH 722	TianJi	RA (10/34) FH (17/35)	RA (33.7 ± 23.6) FH (29.1 ± 22.1)	RA (21.8 ± 3.1) FH (22.4 ± 3.1)
Xin Xiaoming et al. 2023	RCS	IIIb	China	RA 18 FH 22	RA 306 FH 354	TianJi	RA (3/15) FH (5/17)	RA (15.17 ± 1.82) FH (15.59 ± 1.97)	N/A
Xiuyuan Chen et al. 2020	RCS	IIIb	China	RA 31 FH 66	RA 378 FH 786	TianJi	RA(12/19) FH (25/41)	RA (69.8 ± 3.8) FH (69.3 ± 5.1)	RA (24.5 ± 1.9) FH (24.5 ± 2.1)

**Table 2:** Risk of bias of cohort or case–control studies (NOS evaluation)

Study	Selection				Comparability of cohorts	Outcomes			Score
	Representativeness of cohort	Selection of nonexposed cohort	Ascertainment of exposure	Outcome lacking at the beginning		Outcome assessment	Sufficient follow-up time	Follow-up adequacy	
Canglong Hou et al. 2023	*	*	*	N/A	**	*	*	*	8
Chao Li et al. 2022	*	*	*	N/A	**	*	N/A	N/A	6
CHEN Haojie et al. 2021	*	*	*	N/A	**	*	*	*	8
Gabriel S et al.2022	*	*	*	N/A	**	*	N/A	N/A	6
LI Chao et al. 2023	*	*	*	N/A	**	*	N/A	N/A	6
Xin Xiaoming et al. 2023	*	*	*	N/A	**	*	*	*	8
Xiuyuan Chen et al. 2020	*	*	*	N/A	**	*	*	*	8

The total score of NOS evaluation is 9 points

N/A represents that the item has not been scored

* Represents that the item has obtained the score

### Accuracy of pedicle screw-placement

#### Clinically acceptable pedicle screw insertion

**Fig. 2:**
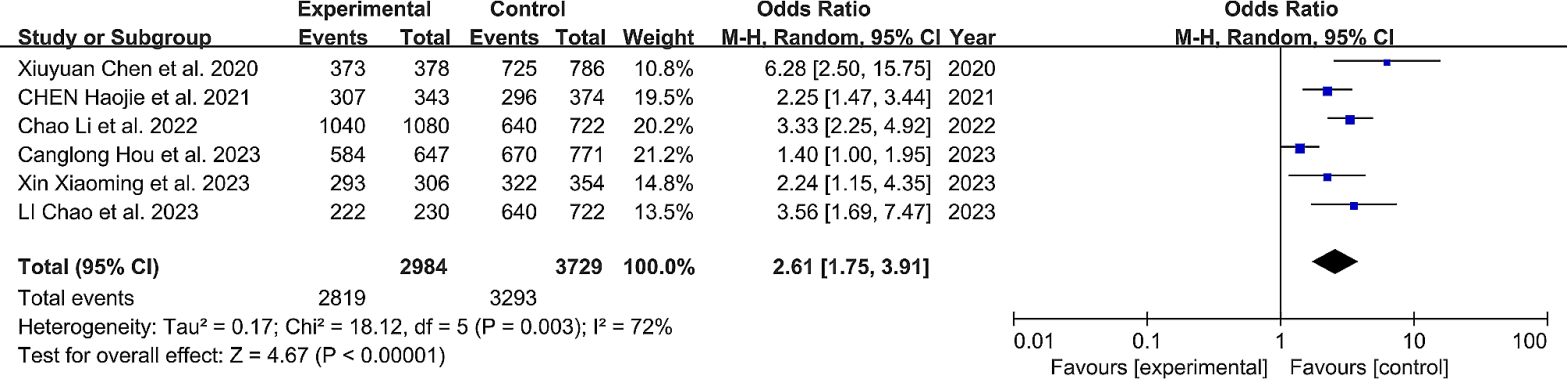
Comparison of clinically acceptable pedicle screw insertion between the robotic-assisted group and the freehand group

When classifying Gertzbein-Robbins grading as A + B or Mobbs-Raley grading as 0 + 1, we categorize it as “clinically acceptable” accuracy. Except for one study [18], all other studies reported on the “clinically acceptable” pedicle screw insertion. Our results confirm that patients undergoing robotic-assisted surgery are 2.61 times more likely to achieve “clinically acceptable” pedicle screw insertion compared to the FH group (OR: 2.61, 95% CI: 1.75–3.91, *P* < 0.0001). (Fig. 2)

#### Perfect pedicle screw insertion

**Fig. 3:**
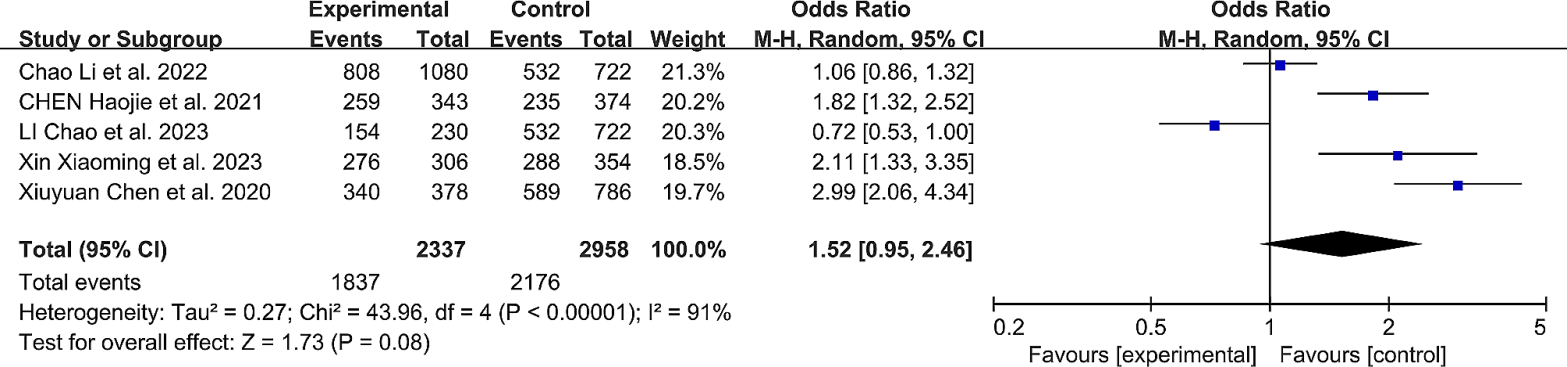
Comparison of perfect pedicle screw insertion between the robotic-assisted group and the freehand group

When classifying Gertzbein-Robbins grading as A or Mobbs-Raley grading as 0, we categorize it as “perfect” accuracy. Except for two studies [11, 18], all studies reported “perfect” pedicle screw insertion. The results indicated that patients undergoing robotic-assisted pedicle screw placement were 1.52 times more likely to achieve “perfect” accuracy compared to the free-hand group, but this result was not statistically significant (OR: 1.52, 95% CI: 0.95–2.46, *P* = 0.08). (Fig. 3)

### Surgical time

**Fig. 4:**
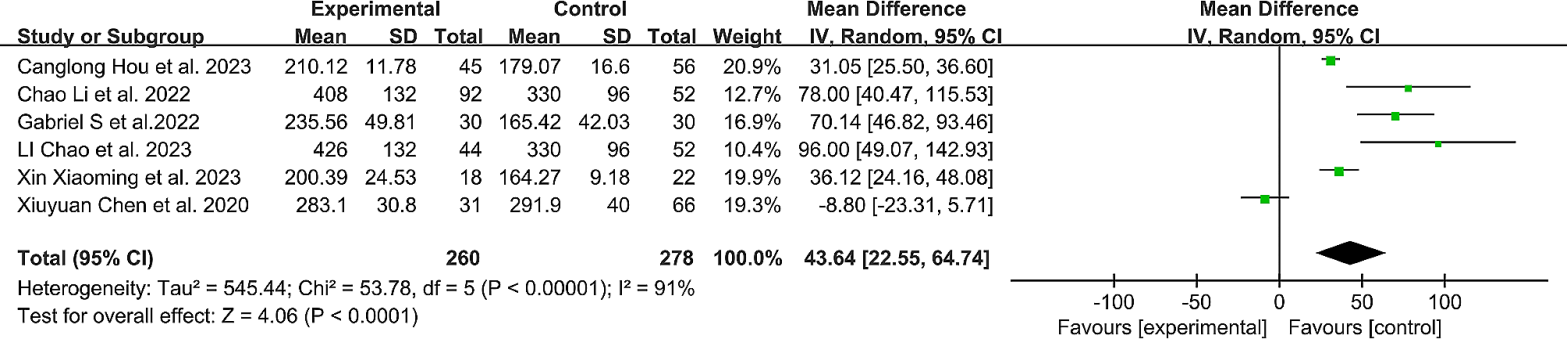
Comparison of surgical time between the robotic-assisted group and the freehand group

A total of 6 studies [7, 11, 16–19] reported surgical time. The results showed a significant difference in surgical time between the robotic-assisted group and the free-hand group (MD: 43.64, 95% CI: 22.25–64.74, *P* < 0.0001), with the robotic group having longer surgical time compared to the free-hand group. (Fig. 4)

### Postoperative hospital stay

**Fig. 5:**
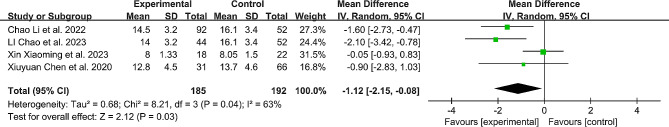
Comparison of postoperative hospital stay between the robotic-assisted group and the freehand group

Four studies [7, 16, 17, 19] reported postoperative hospital stay for both the robotic-assisted and free-hand groups. The results showed that the average postoperative hospital stay in the robotic group was shorter than that in the free-hand group, and the difference between the groups was statistically significant (MD: -1.12, 95% CI: -2.15 to -0.08, *P* = 0.03). (Fig. 5)

### Complications

**Fig. 6:**
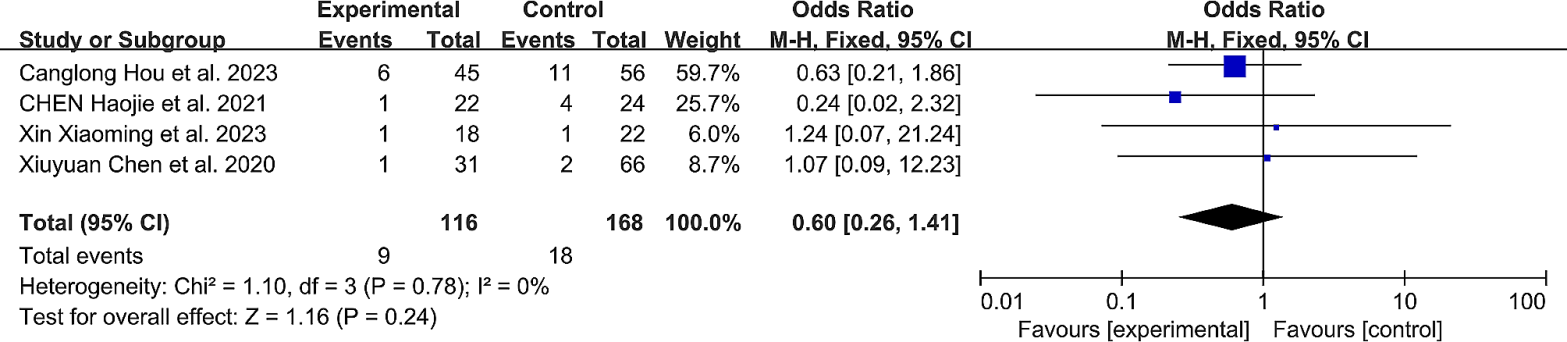
Comparison of complications between the robotic-assisted group and the freehand group

Four included studies [11, 12, 16, 17] reported complications after pedicle screw insertion in both the robotic-assisted group (9 patients, 7.75%) and the free-hand group (18 patients, 10.71%). Complications included screw loosening, surgical revisions, wound infections, and nerve root injuries. Compared to patients with free-hand screw placement, the overall incidence of complications in patients with robotic-assisted screw placement was reduced by 27.6%, but the difference between the two groups was not statistically significant (OR: 0.60, 95% CI: 0.26–1.41, *P* = 0.24). (Fig. 6; Table 3)

**Table 3:** Complications and the number of cases in the included studies

Complication	Study	RA	FH
poor wound healing	CHEN Haojie et al. 2021	1	2
	Xin Xiaoming et al. 2023	1	1
nerve root injury	CHEN Haojie et al. 2021		2
screw loosening	Canglong Hou et al. 2023	2	4
adding on	Canglong Hou et al. 2023		3
proximal junctional kyphosis	Canglong Hou et al. 2023	3	4
revision	Canglong Hou et al. 2023	1	
postural headache	Gabriel S et al.2022		1
syncope	Gabriel S et al.2022		1
pressure sores	Xiuyuan Chen et al. 2020	1	
dural laceration	Xiuyuan Chen et al. 2020		2

### Blood loss

**Fig. 7:**
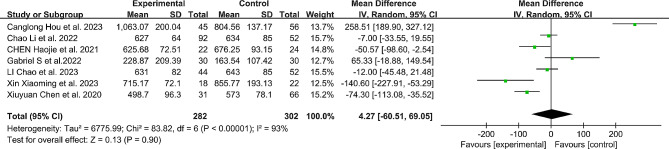
Comparison of blood loss between the robotic-assisted group and the freehand group

A total of 7 studies [7, 11, 12, 16–19] reported intraoperative blood loss for both the robotic and free-hand groups. However, there was no significant difference between the two groups in terms of intraoperative blood loss (MD: 4.27, 95% CI: -60.51 to 69.05, *P* = 0.90). (Fig. 7)

**Fig. 8:**
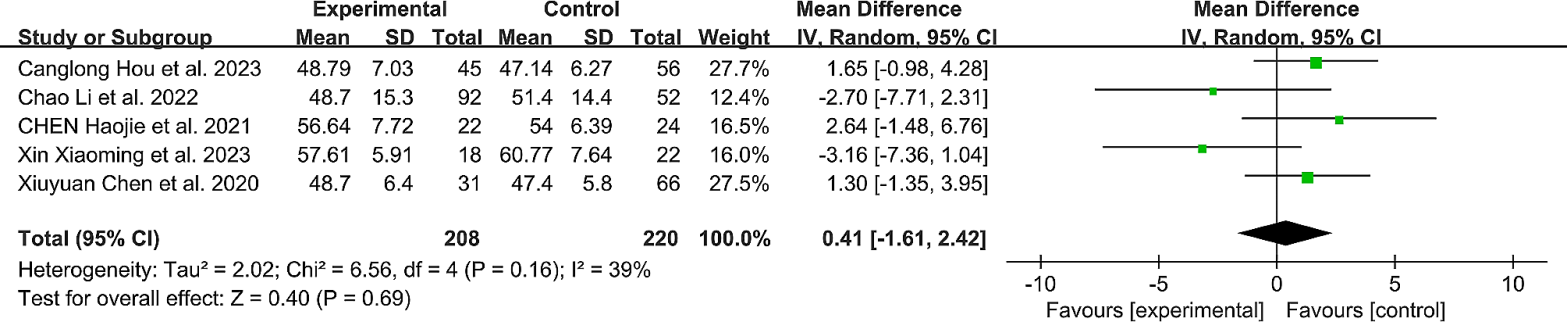
Comparison of preoperative cobb angle between the robotic-assisted group and the freehand group

**Fig. 9:**
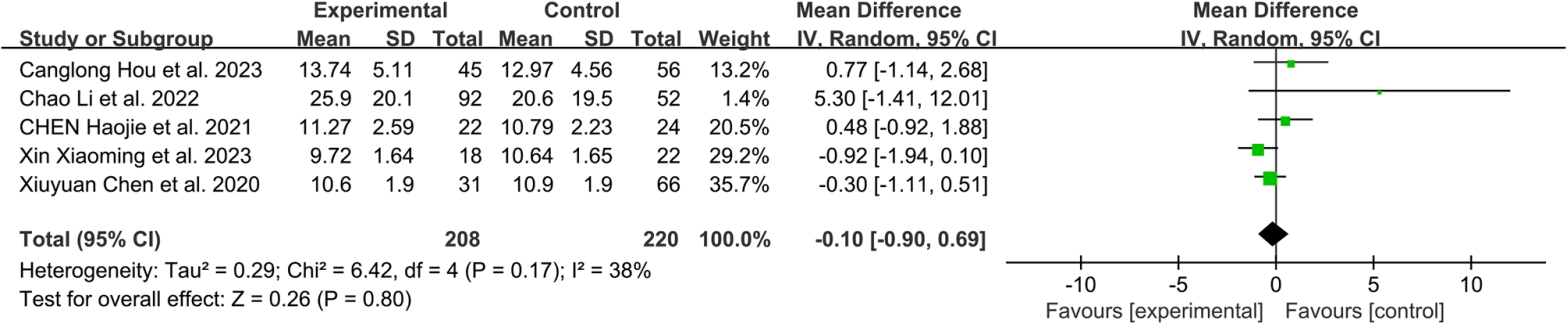
Comparison of postoperative cobb angle between the robotic-assisted group and the freehand group

Cobb Angle:

A total of 5 studies reported the preoperative and postoperative Cobb Angle changes in detail between the RA group and the FH group. The results showed that there was no significant difference in the preoperative Cobb Angle between the RA group and the FH group (MD: 0.41, 95%CI: -1.61-2.42, *P* = 0.69) (Fig. 8). At the same time, there was no statistically significant difference in Cobb Angle between the two groups after operation (MD: -0.1, 95%CI: -0.90-0.69, *P* = 0.80) (Fig. 9).

## Discussion

Scoliosis is a 3D deformity often characterized by narrow pedicles, abnormal vertebral rotation, and abnormalities in the vertebral body, increasing the risk of misplacement of the pedicle screw. Kwan et al. [21] reported pedicle screw misplacement rates ranging from 5 to 41% in lumbar spine surgeries, and from 3 to 51% in thoracic spine surgeries. Screw misplacement can cause severe neurovascular complications, and revision surgery for scoliosis correction can be challenging [22]. Despite the implementation of robust preventive techniques such as intraoperative radiography, computed tomography, and neurophysiological monitoring [23] in spinal surgeries, screw-related complications still pose significant challenges to the safety and patient satisfaction of spinal deformity correction. Recently, the development of robot-assisted technology has brought new advancements to scoliosis surgery.

In recent decades, the rapid development of robotic surgery has increased the number of spinal surgeries being performed. Mazor SpineAssist (Mazor Surgical Technologies, Caesarea, Israel), approved by the United States Food and Drug Administration (FDA) in 2004, was the first FDA-approved spinal surgery robot in the United States [8, 24]. Since then, several spinal robotic systems have been developed, including Mazor Robotics, Orthbot [25, 26], and TiRobot [27, 28]. Moreover, in the past decade, new robotic platforms called ExcelsiusGPS® [29] have emerged that integrate semiautomated robotics with real-time 3D navigation technology. Innovations in surgical robotics combined with computer-based intraoperative navigation systems, are driving advances in modern spinal surgery. Robot-assisted screw placement technology is used globally in various spinal surgeries. In 2023, Matur et al. [30] concluded through a meta-analysis that robot-assisted and navigation systems are safer and more effective than freehand placement in thoracolumbar surgery. Hou et al. [11] and Linden et al. [18] reported that robot-assisted technology achieved better accuracy than freehand placement in patients with adolescent idiopathic scoliosis (AIS) patients. However, these studies had limited case numbers, and there is still insufficient high-quality research on the accuracy of pedicle screw insertion with robotic assistance in patients with spinal scoliosis and reliable conclusions could not be drawn. We conducted this meta-analysis to compare the accuracy and postoperative effectiveness of robot-assisted screw placement technology and freehand placement technology in scoliosis surgery.

Our findings indicated significant statistical differences between the two groups in terms of radiographic and clinical outcomes. The robot-assisted group demonstrated a higher rate of “clinically acceptable” accuracy (*P* < 0.00001) than that of the freehand group, whereas there was no difference in the accuracy of perfect screw placement (*P* = 0.08) between the two groups. Robot-assisted technology enables 3D visualization of spinal images for preoperative planning, allowing for preplanning of screw diameter, length, and placement angles [31]. In addition, the stability of the mechanical arm reduces human error [32]. Adequate preoperative and intraoperative registration is crucial for screw placement accuracy and surgical duration [33]. In this meta-analysis, the TiRobot used in five studies [7, 12, 16, 17, 19]was registered using intraoperative CT scans, while the Mazor robot used in two studies [11, 18] was registered using preoperative CT scans. Integrating intraoperative X-rays with preoperative CT scans poses a challenge. The Mazor Renaissance robot initially inputs preoperative CT scan data into the software to determine screw size and position. Subsequently, it uses two intraoperative X-ray images to identify the position of each vertebral segment. The robotic device then moves to the planned screw trajectory and direction to complete the integration process. Furthermore, intraoperative image matching and alignment have also been addressed. Systems such as the ROSA Spine Robot [34] and TiRobot [28] utilize optical tracking and real-time end-effector compensation to monitor patient movement during respiration and surgical maneuvers and make adjustments accordingly.

Compared with the freehand group, no significant differences were observed in the perfect screw placement rate. We attribute this to the relatively narrow pedicles in patients with spinal deformities, particularly those with AIS, and abnormal rotation of the vertebral bodies, which makes perfect screw placement challenging. Furthermore, rotational errors of the vertebral bodies and limited rotation of the mechanical arm combined with muscle-related disturbances can lead to image changes and errors during preoperative planning, as suggested by Li et al [7]. This issue has seen advancements with the development of technology. For instance, the third-generation Mazor X features increased arm extension and strength [34]. Additionally, the ExcelsiusGPS® robotic arm is designed to be rigid, maintaining a deflection of less than 1.0 mm under a lateral force of 200 N [34]. It is also equipped with a sensor that can detect excessive lateral forces on the instrument holder in real time. Additionally, to improve the accuracy and safety of scoliosis surgery, both robotic-assisted screw placement and computer-assisted navigation are employed. A study by Al-Naseem et al. [35] compared the effectiveness of robotic-assisted screw placement versus standalone navigation in scoliosis surgeries. The results indicated that robotic-assisted surgery had a significant advantage in the accuracy of pedicle screw placement. However, the downside of robotic-assisted surgery is the significantly longer operative time. There were no significant differences in other clinical outcome measures or radiation exposure, and the cost of robotic-assisted surgery cannot be overlooked. Further research is needed to validate these findings. Therefore, robot-assisted technology, through preoperative planning and real-time monitoring, combined with the stability of the robotic arm, simplicity of operation, and good repeatability, enables less experienced surgeons to perform scoliosis surgery with the assistance of the robot. In contrast, novice surgeons using freehand techniques for scoliosis surgery often find it more difficult due to abnormal pedicle and vertebral rotation, which requires a longer learning curve to master.

In our findings, we observed no statistically significant difference in preoperative and postoperative Cobb angles between the robot-assisted and freehand groups. Therefore, we did not find any difference in Cobb angle correction rates between the two groups. We posit that this might be attributed to optimal screw placement points planned by the robotic system, potentially hindering smooth rod passage. Consequently, surgeons may have opted for suboptimal trajectories, resulting in less-than-optimal correction outcomes. Further research is warranted to delineate the influence of both cohorts on Cobb angle correction rates. Currently, deformity planning software has been integrated into robotic technology. In a prospective study by K. Khalifeh et al. [36], preoperative planning software (Mazor X-Align) for Mazor X Robotics was used to plan surgery, and the final results indicated that the system’s predictive accuracy was within 6° and 9° in the coronal and sagittal planes, respectively. With technological advancements, such software combined with robotic technology can plan screw entry points to facilitate rod passage and incorporate expected changes in alignment based on osteotomy location. Whether the combination of such planning software with robotic technology can improve Cobb angle correction rates while maintaining good screw placement accuracy remains to be further studied.

Patients with spinal deformities, even minor cortical or pedicle violations, may experience severe complications, such as nerve or vascular injuries [37]. Our study found no significant differences in complications (*P* = 0.24) or blood loss (*P* = 0.90) between the groups using robots. No severe complications were observed in either of the groups. This may be attributed to the relatively small number of cases in our study and the predominance of adolescent idiopathic scoliosis (AIS) patients and patients with degenerative scoliosis among the included cases. While our study covered various types of spinal curvature, including idiopathic, congenital, neuromuscular, and degenerative scoliosis, the majority of cases comprised AIS and degenerative scoliosis patients. Compared to those with severe spinal deformities, these patients generally undergo less complex surgeries and experience fewer postoperative complications. The lack of a difference in intraoperative blood loss between the two groups was not surprising. We believe that, unlike typical thoracolumbar procedures, spinal deformity surgeries involve significant differences in osteotomy, correction, and fusion segments, leading to noticeable differences in intraoperative blood loss. Fatima et al. [38] concluded in a meta-analysis that robot-assisted screw placement resulted in less blood loss in spinal surgeries than in the freehand group, attributing this to the minimally invasive nature of robotic surgeries. We believe that the high accuracy of screw placement and the minimally invasive nature of robotic surgeries may have led to fewer complications and less blood loss in the robot-assisted group. As more related studies are published and the number of cases increases, further research is needed to confirm whether the robotic approach outperforms the freehand approach in terms of complications and blood loss.

Similar to previous meta-analyses, our study found that the robot-assisted group had longer surgical durations (*P* < 0.001) than the freehand group. Chen et al. [17] suggested that multiple scans and planning are typically required for long-segment deformity surgeries, which may increase the surgical duration. Conversely, Hyun et al. [39] reported that the screw-insertion time decreased by 1.5 min between the first 15 cases and the subsequent 15 cases of robot-assisted surgeries. As the total number of robot-assisted surgical cases increased, the prolonged surgical duration appeared to decrease, possibly due to the learning curve of robotics [40]. Our meta-analysis results showed shorter hospital stays (*P* < 0.05) in the robotic group, which was attributed to the higher accuracy of screw placement and the relatively minimally invasive nature of robotic surgeries. Despite the lack of significant differences in complication rates between the two groups, the clinical acceptability of screw placement was significantly higher in the robotic-assisted group compared to the freehand group. The lower accuracy of screw placement in the freehand group may necessitate extended postoperative bed rest to monitor for complications, thereby preventing serious issues that could arise from early mobilization. However, this finding requires further research for validation. We believe that owing to the learning curve, surgical duration, intraoperative blood loss, and hospital stay may decrease as the number of cases and experience increases.

Our meta-analysis results indicate that compared to conventional freehand techniques, robot-assisted screw placement technology significantly improves “clinically acceptable” accuracy rates and shortens hospital stays, but it also prolongs surgery duration. There were no significant differences in perfect screw placement rates, postoperative complications, or intraoperative blood loss. Like other meta-analyses, we conducted a cautious analysis and interpretation of these results. In summary, we believe that robot-assisted screw placement in scoliosis surgery can improve screw accuracy and lead to better postoperative clinical outcomes. However, this viewpoint requires further validation. At the same time, we must recognize the drawbacks of robot-assisted screw placement. Firstly, although some studies have reported intraoperative radiation doses, the radiation dose from preoperative CT scans used for image acquisition and registration remains unknown. Secondly, given the high economic costs of robotic surgery and equipment maintenance, the widespread adoption and promotion of robotic technology remain challenging.

This study had some limitations. First, our meta-analysis included only seven retrospective cohort studies (RCS), with a small number of articles and a lack of prospective studies. The studies included in this review were summarized and listed to allow surgeons to carefully examine and evaluate their limitations and biases. Second, different types of robots, such as Mazor and Tianji, were included in this study. However, owing to the limited number of included studies, it was not possible to compare the impact of different robots on the outcomes. Radiation exposure and cost issues have always been important topics of concern in robot-assisted screw placement technology [41]. Owing to the limited and heterogeneous RCS data, it was not possible to assess patient-reported radiation exposure and costs. Prospective randomized studies are needed to further compare the safety and postoperative outcomes of this technology in scoliosis surgery. Additionally, because of the different types of scoliosis, comparisons between the two groups should be based on the type of scoliosis. Unfortunately, this was not possible because of insufficient data. Finally, two studies included in this meta-analysis combined robotic technology with computer navigation technology, which may have influenced the results.

## Conclusion

This meta-analysis indicated that compared with conventional freehand techniques, robot-assisted screw placement technology significantly improves accuracy and reduces hospital stay, but prolongs surgical duration, with no significant differences in perfect screw placement rate, postoperative complications, or intraoperative blood loss. However, there were potential confounding factors in our study, and the number of cases was limited. Intraoperative radiation exposure, costs, and long-term postoperative outcome indicators were not analyzed. Therefore, further research is needed to provide additional relevant information on short- and long-term clinical outcomes to validate these findings. Although these findings require further validation through extensive research, we believe that in scoliosis surgery, particularly for complex cases, robot-assisted pedicle screw placement offers superior accuracy and postoperative clinical outcomes compared to freehand placement.

### Electronic supplementary material

Below is the link to the electronic supplementary material.

Supplementary Material 1

## Data Availability

No datasets were generated or analysed during the current study.

## Abbreviations

AIS: adolescent idiopathic scoliosis
CI: confidence interval
FH: freehand
MD: Mean Difference
NOS: Newcastle–Ottawa Scale
OR: Odds Ratio
PCS: prospective cohort studies
RA: robot-assisted
RCS: retrospective comparative studies
Freehand: RCT, randomized controlled trial

## References

[1] Cheung ZB (2019). Idiopathic scoliosis in children and adolescents: emerging techniques in Surgical Treatment. World Neurosurg.

[2] Matsumoto M (2014). Updates on surgical treatments for pediatric scoliosis. J Orthop Sci.

[3] Sarwahi V (2014). Pedicle screws adjacent to the great vessels or viscera: a study of 2132 pedicle screws in pediatric spine deformity. J Spinal Disord Tech.

[4] Fujimori T (2014). Safety of pedicle screws and spinal instrumentation for pediatric patients: comparative analysis between 0- and 5-year-old, 5- and 10-year-old, and 10- and 15-year-old patients. Spine (Phila Pa 1976).

[5] Liljenqvist UR, Halm HF, Link TM (1997). Pedicle screw instrumentation of the thoracic spine in idiopathic scoliosis. Spine (Phila Pa 1976).

[6] Zhao Y (2020). Risk factors related to Superior Facet Joint violation during lumbar Percutaneous Pedicle Screw Placement in minimally invasive transforaminal lumbar Interbody Fusion (MIS-TLIF). World Neurosurg.

[7] Li C et al. Comparison of the Accuracy of Pedicle Screw Placement using a fluoroscopy-assisted free-hand technique with robotic-assisted Navigation using an O-Arm or 3D C-Arm in scoliosis surgery. Global Spine J, 2022: p. 21925682221143076. 10.1177/21925682221143076 PMC11289529 36455162

[8] D’Souza M (2019). Robotic-assisted spine surgery: history, efficacy, cost, and Future trends. Robot Surg.

[9] Yu CC (2022). Propensity-matched comparison of 90-Day complications in robotic-assisted Versus non-robotic assisted lumbar Fusion. Spine (Phila Pa 1976).

[10] Lee NJ (2021). Do robot-related complications influence 1 year reoperations and other clinical outcomes after robot-assisted lumbar arthrodesis? A multicenter assessment of 320 patients. J Orthop Surg Res.

[11] Hou C (2022). Comparison of robot versus fluoroscopy-assisted pedicle screw instrumentation in adolescent idiopathic scoliosis surgery: a retrospective study. Front Surg.

[12] Chen H (2021). [Study on robot-assisted pedicle screw implantation in adolescent idiopathic scoliosis surgery]. Zhongguo Xiu Fu Chong Jian Wai Ke Za Zhi.

[13] Panic N (2013). Evaluation of the endorsement of the preferred reporting items for systematic reviews and meta-analysis (PRISMA) statement on the quality of published systematic review and meta-analyses. PLoS ONE.

[14] Goz V (2013). Perioperative complications and mortality after spinal fusions: analysis of trends and risk factors. Spine (Phila Pa 1976).

[15] Raley DA, Mobbs RJ (2012). Retrospective computed tomography scan analysis of percutaneously inserted pedicle screws for posterior transpedicular stabilization of the thoracic and lumbar spine: accuracy and complication rates. Spine (Phila Pa 1976).

[16] Xin X (2023). Application of orthopedic robot-assisted screw placement in the correction of adolescent idiopathic scoliosis. Chin J Tissue Eng Res.

[17] Chen X (2020). Robot-assisted orthopedic surgery in the treatment of adult degenerative scoliosis: a preliminary clinical report. J Orthop Surg Res.

[18] Linden GS et al. Pedicle Screw Placement in adolescent idiopathic scoliosis: a comparison between Robotics coupled with Navigation versus the Freehand technique. Sens (Basel), 2022. 22(14). 10.3390/s22145204 PMC9316760 35890882

[19] Chao LX, Hao SUN, Yonghao LI, Suomao TIAN, Xinyu YUAN, Lianlei LIU (2023). Clinical application of robotic-assisted navigation based on 3D C-arm in 44 cases of scoliosis surgery. J Shandong University(Health Sciences).

[20] Stang A (2010). Critical evaluation of the Newcastle-Ottawa scale for the assessment of the quality of nonrandomized studies in meta-analyses. Eur J Epidemiol.

[21] Kwan MK (2017). Accuracy and Safety of Pedicle Screw Placement in adolescent idiopathic scoliosis patients: a review of 2020 screws using computed Tomography Assessment. Spine (Phila Pa 1976).

[22] Sponseller PD (2010). Pediatric revision spinal deformity surgery: issues and complications. Spine (Phila Pa 1976).

[23] Newell R (2018). An intraoperative fluoroscopic method to accurately measure the post-implantation position of pedicle screws. Int J Comput Assist Radiol Surg.

[24] Kim HJ et al. *Comparative study of 1-year clinical and radiological outcomes using robot-assisted pedicle screw fixation and freehand technique in posterior lumbar interbody fusion: A prospective, randomized controlled trial* Int J Med Robot, 2018. 14(4): p. e1917. 10.1002/rcs.1917 29786165

[25] Li Z (2020). A preliminary study of a novel robotic system for pedicle screw fixation: a randomised controlled trial. J Orthop Translat.

[26] Li J (2021). Evaluation of a new spinal surgical robotic system of Kirschner wire placement for lumbar fusion: a multi-centre, randomised controlled clinical study. Int J Med Robot.

[27] Feng S, Tian W, Wei Y (2020). Clinical effects of oblique lateral Interbody Fusion by Conventional Open versus Percutaneous Robot-assisted minimally Invasive Pedicle Screw Placement in Elderly patients. Orthop Surg.

[28] Han X et al. Safety and accuracy of robot-assisted versus fluoroscopy-assisted pedicle screw insertion in thoracolumbar spinal surgery: a prospective randomized controlled trial. J Neurosurg Spine, 2019: p. 1–8. 10.3171/2018.10.SPINE18487 30738398

[29] Jiang B (2018). Pedicle screw accuracy assessment in ExcelsiusGPS® robotic spine surgery: evaluation of deviation from pre-planned trajectory. Chin Neurosurg J.

[30] Matur AV (2023). Robotic and navigated pedicle screws are safer and more accurate than fluoroscopic freehand screws: a systematic review and meta-analysis. Spine J.

[31] Lonjon N (2016). Robot-assisted spine surgery: feasibility study through a prospective case-matched analysis. Eur Spine J.

[32] Lee NJ (2022). A multicenter study of the 5-year trends in robot-assisted spine surgery outcomes and complications. J Spine Surg.

[33] Overley SC (2017). Navigation and Robotics in spinal surgery: where are we now?. Neurosurgery.

[34] Huang M (2021). The current state of navigation in robotic spine surgery. Ann Transl Med.

[35] Al-Naseem AO, et al. Robot-assisted pedicle screw insertion versus navigation-based and freehand techniques for posterior spinal fusion in scoliosis: a systematic review and meta-analysis. Spine Deform; 2024. 10.1007/s43390-024-00879-y PMC11343815 38619784

[36] Khalifeh K (2024). Spinal Robotics in adult spinal deformity surgery: a systematic review. Neurospine.

[37] Rajasekaran S (2007). Randomized clinical study to compare the accuracy of navigated and non-navigated thoracic pedicle screws in deformity correction surgeries. Spine (Phila Pa 1976).

[38] Fatima N (2021). Safety and accuracy of robot-assisted placement of pedicle screws compared to conventional free-hand technique: a systematic review and meta-analysis. Spine J.

[39] Hyun SJ (2017). Minimally invasive robotic Versus Open Fluoroscopic-guided spinal instrumented fusions: a Randomized Controlled Trial. Spine (Phila Pa 1976).

[40] Ghasem A (2018). The arrival of Robotics in spine surgery: a review of the literature. Spine (Phila Pa 1976).

[41] Menger RP (2018). A cost-effectiveness analysis of the Integration of Robotic Spine Technology in spine surgery. Neurospine.

