# Supplementary material for: Accuracy and postoperative assessment of robot-assisted placement of pedicle screws during scoliosis surgery compared with conventional freehand technique: a systematic review and meta-analysis
Source: J Orthop Surg Res. 2024 Jun 20;19:365. doi: 10.1186/s13018-024-04848-z (PMC11188284; doi:10.1186/s13018-024-04848-z)
Supplement: Supplementary file 1 — Supplementary Material 1 [file 13018_2024_4848_MOESM1_ESM.docx]

**Title**

**Accuracy and Postoperative Assessment of Robot-Assisted Placement of Pedicle Screws during Scoliosis Surgery Compared with Conventional Freehand Technique: A Systematic Review and Meta-Analysis**

**Authors**

Wei Cui MD*1 , Xinglin Liu MD*2, Zhiheng Zhao MD2 , Zihe Feng MD1, Xianglong Meng#2,

* Wei Cui, Xinglin Liu contribute equally to this study

**Affiliations**

1Department of Orthopedic Surgery, Beijing Chaoyang Hospital, Capital Medical University of China

2Department of Orthopaedic Surgery, Beijing Anzhen Hospital, Capital Medical University of China

**^#^Corresponding authors**

Xianglong Meng

Department of Orthopedic Surgery, Beijing AnZhen Hospital, Capital Medical University, No. 2 Anzhen Road, Chaoyang District, Beijing, 100029, China

E-mail: [spinesurgeonmeng@ccmu.edu.cn](mailto:spinesurgeonmeng@ccmu.edu.cn)

Tel&Fax +86-13811618995
